# Hemodialysis catheter-related infection caused by *Pannonibacter phragmitetus*: a rare case report in China

**DOI:** 10.3389/fcimb.2022.926154

**Published:** 2022-07-22

**Authors:** Ruizhi Tang, Jing Wang, Yu Zhan, Kaifu Wu, Hui Wang, Zhongxin Lu

**Affiliations:** ^1^ Department of Medical Laboratory, The Central Hospital of Wuhan, Tongji Medical College, Huazhong University of Science and Technology, Wuhan, China; ^2^ Key Laboratory for Molecular Diagnosis of Hubei Province, The Central Hospital of Wuhan, Tongji Medical College, Huazhong University of Science and Technology, Wuhan, China; ^3^ Department of Radiology, The Central Hospital of Wuhan, Tongji Medical College, Huazhong University of Science and Technology, Wuhan, China; ^4^ Cancer Research Institute of Wuhan, The Central Hospital of Wuhan, Tongji Medical College, Huazhong University of Science and Technology, Wuhan, China

**Keywords:** *Pannonibacter phragmitetus*, hemodialysis, catheter infection, recurrent fever, anti-infective treatment

## Abstract

*Pannonibacter phragmitetus* (*P. phragmitetus*) is rarely related with human disease. We reported a case of catheter-related infection caused by *P. phragmitetus* in a 68-year-old woman on hemodialysis. The patient developed recurrent fever during hemodialysis and blood cultures were positive for *P. phragmitetus*. The patient’s body temperature returned to normal after intravenous cefoperazone/sulbactam treatment, and the hemodialysis catheter was locked with gentamicin and urokinase. The potential anti-infective treatment against *P. phragmitetus* was discussed.

## Introduction

*Pannonibacter phragmitetus* (*P. phragmitetus*) is a gram-negative rod, which is recognized from a Hungarian soda lake in 2003 (Borsodi et al., 2003). *P. phragmitetus* is commonly applied to environmental pollution control due to its ability to remove nitrogen and metals in water and soil (Bai et al., 2019; Chai et al., 2019; Wang et al., 2019; Liao et al., 2020; Saravanan et al., 2021). Although it was first identified as *Achromobacter* groups B and E from human blood (Holmes et al., 1990; Holmes et al., 1993; Holmes et al., 2006), little is known about its pathogenicity. After searching in PubMed and Web of Science, we found only 4 cases of infection caused by *P. phragmitetus*: one case of late replacement valve endocarditis (McKinley et al., 1990), one case of recurrent septicemia (Jenks and Shaw, 1997), one case of liver abscess (Wang et al., 2017), and one case of bacteriemia (Gallardo et al., 2020). Here, we presented probably the first case of hemodialysis catheter infection caused by *P. phragmitetus* in China.

## Case presentation

A 68-year-old woman with end-stage renal disease was admitted to our hospital for hemodialysis on September 16, 2021. The patient had a history of left kidney cyst, atrophy of both kidneys, chronic kidney disease, obstructive pulmonary disease, and hypertension. A rectal resection was performed in 2002. Her normal medication consisted of nifedipine (20 mg, bid), benazepril (10 mg, bid) and terazosin (2 mg, tid). Since January 19, 2021, the patient started hemodialysis three times a week *via* a catheter in the right internal jugular vein at a local hospital. On August 17, the patient had chills during hemodialysis. After returning home, she developed fever and sweating with a body temperature of 39.1°C. Her body temperature returned to normal after she received diclofenac and anti-infective treatment. On September 1, she became febrile again after hemodialysis. She was hospitalized at the local hospital for 10 days and she was recovered after receiving anti-infective therapy. On September 13, the patient developed chills, fever and sweating again with a body temperature of 38.6°C during hemodialysis. Her body temperature returned to normal after physical cooling.

Upon admission, the patients’ body temperature was 36.9°C, pulse rate was 82 beats/min, respiratory rate was 18 breaths/min, and blood pressure was 200/110 mmHg. Laboratory tests (see **Table 1**) showed impaired kidney function (CO_2_ 21.4 mmol/L, creatinine 958.3 μmol/L, uric acid 460 μmol/L, BUN 23.2mmol/L), anemia (RBC 3.29×10^12^/L, Hb 102 g/L), vitamin D deficiency (28.11 nmol/L), and hyperparathyroidism (PTH 226.1 pg/mL).

**Table 1:** Abnormal diagnostic results.

Parameter	Values	Normal range
RBC (×10^12^/L)	3.29	3.8~5.1
Hb (g/L)	102	115~150
HCT (%)	31.1	35~45
CO_2_ (mmol/L)	21.4	22~29
Creatinine (μmol/L)	958.3	41~81
Uric acid (μmol/L)	460	155~357
BUN (mmol/L)	23.2	2.76~8.07
AST (U/L)	11	13~35
Calcium (mmol/L)	2.02	2.2~2.55
Phosphorus (mmol/L)	1.59	0.85~1.51
PTH (pg/mL)	226.1	12~88
Vitamin D (nmol/L)	28.11	>75
PCT (ng/ml)	1.04	<0.046

RBC, red blood cell; Hb, Hemoglobin; HCT, Hematocrit; BUN, blood urea nitrogen; AST (aspartate aminotransferase), PTH, parathyroid hormone; PCT, procalcitonin.

**Figure 1:**
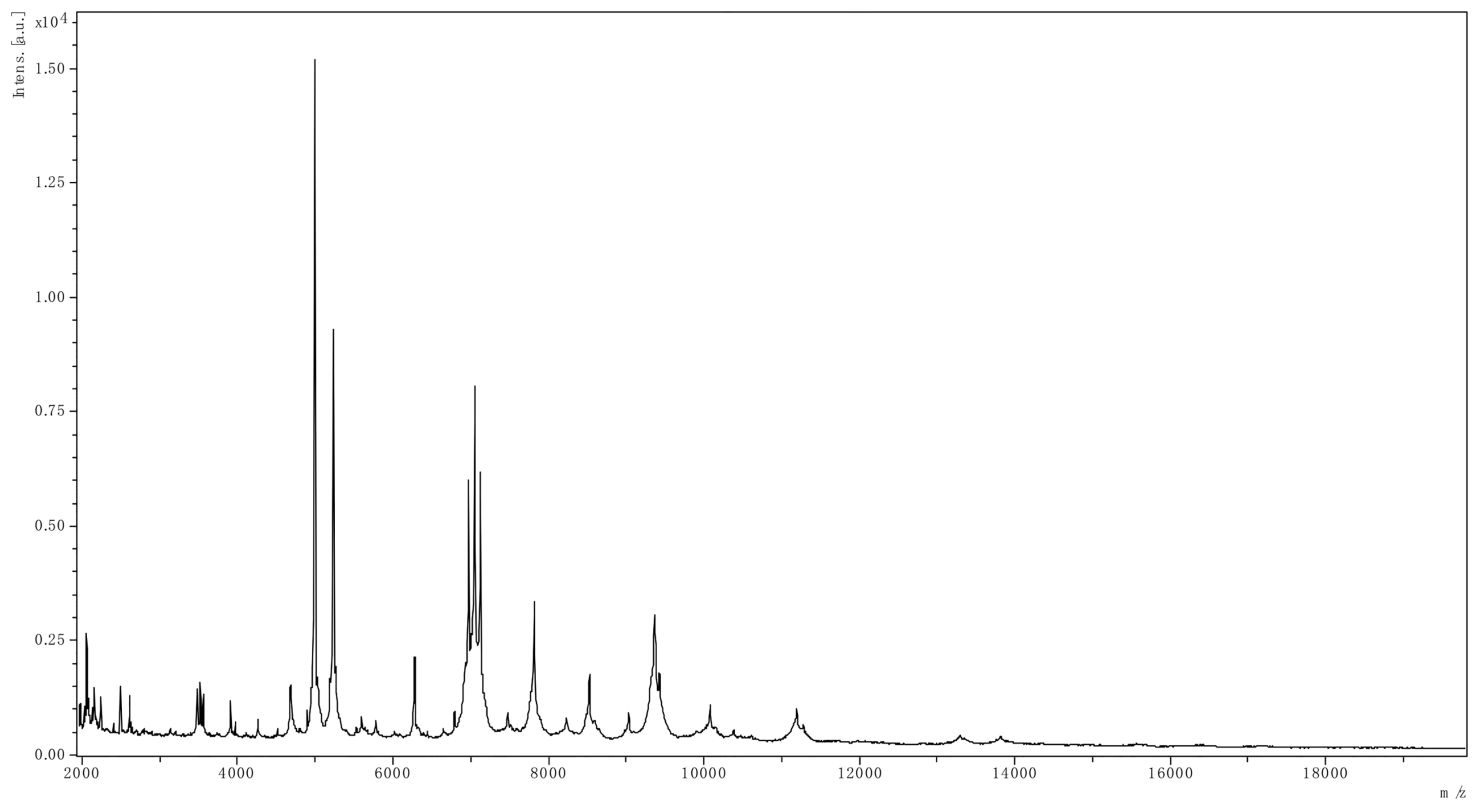
The spectrum of *P. phragmitetus* analyzed by MALDI Biotyper 3.1 (Bruker Daltonik GmbH, Germany).

On the next day of admission, the patient developed fever during hemodialysis, and she was treated with intravenous demethylvancomycin (400 mg, qd). A pair of venous blood samples were collected prior to antimicrobial therapy, as well as a pair of blood sample drawn from the catheter. Aerobic blood cultures were positive after 2 days of incubation (48 h 25 min). A gram staining revealed gram-negative rods. Positive cultures were then transferred to Columbia blood agar plates, MacConkey agar plates, chocolate agar plates and Sabouraud’s agar plates at 35°C, and Sabouraud’s agar plates at 28°C. After incubation overnight, *P. phragmitetus* (**Figure 1**) was identified by matrix-assisted laser desorption ionization/time of flight mass spectrometry (LVD MALDI Biotyper System, Bruker Daltonik GmbH, Germany). *P. phragmitetus* was also verified by DNA sequencing (**Supplementary Figure 1 and Data Sheet 1**), using bacterial 16S rDNA primers (forward primer: 5’-AGTTTGATCMTGGCTCAG-3’, reverse primer: 5’-GGTTACCTTGTTACGACTT-3’). Antimicrobial susceptibility test (AST) was performed by the Kirby-Bauer disc diffusion method (Thermo Fisher Scientific, USA). The minimum inhibitory concentration (MIC) of *P. phragmitetus* for antibiotics were determined by BD Phoenix 100 system using NMIC/ID-4 susceptibility panels (**Table 2**). *P. phragmitetus* was sensitive to cefoxitin, imipenem, meropenem, amikacin, gentamicin, tetracycline, minocycline, ciprofloxacin, levofloxacin, chloramphenicol, but resistant against penicillin, ampicillin, piperacillin, piperacillin/tazobactam, aztreonam, trimethoprim/sulfamethoxazole, vancomycin, teicoplanin, clindamycin and linezolid amine. The patient was then changed to intravenous cefoperazone/sulbactam (500mg/500mg, bid) treatment. The hemodialysis catheter was locked with gentamicin (1.8×10^4^ IU) and urokinase (1×10^5^ IU).

**Table 2:** Antimicrobial susceptibility of *P. phragmitetus*.

Antimicrobial agent	MIC (μg/ml)	Interpretation
Amikacin	<=8	S
Gentamicin	<=2	S
Imipenem	<=1	S
Meropenem	<=1	S
Cefazolin	>16	R
Cefotaxime	2	I
Aztreonam	>16	R
Ampicillin	>16	R
Piperacillin	>64	R
Amoxicillin/Clavulanate potassium	<=4/2	R
Ampicillin/Sulbactam	>16/8	R
Clindamycin	>2	R
Chloromycetin	<=4	S
Ciprofloxacin	<=0.5	S
Levofloxacin	<=1	S
Tetracycline	<=1	S

MIC, the minimum inhibitory concentration.

**Figure 2:**
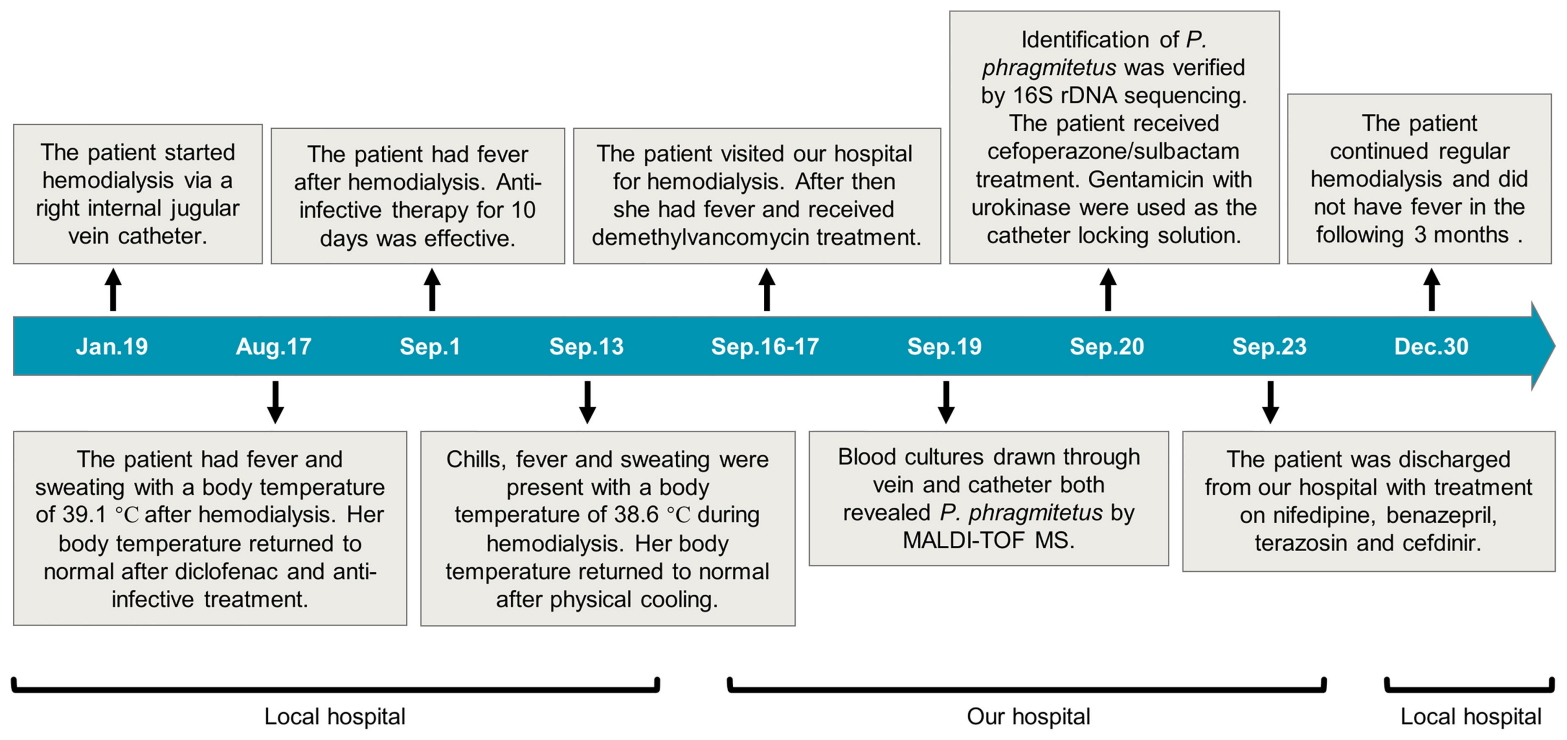
The patient’s medical timeline.

Although clinical results were improved, but blood tests still showed a sign of infection (PCT 1.04 ng/ml, see **Table 1**). However, the patient refused to replace the hemodialysis catheter. On September 23, the patient was discharged from our hospital with treatment on nifedipine (20 mg, bid), benazepril (10 mg, bid), terazosin (2 mg, tid) and cefdinir (100 mg, tid). The patient underwent regular hemodialysis at the local hospital and developed no fever in the following 3 months (the patient’s medical timeline was shown in **Figure 2**).

## Discussion

The first case of infection caused by *P. phragmitetus* was reported in a male patient who developed a low-grade fever in 1988 (McKinley et al., 1990). The patient underwent pulmonary autograft replacement in 1987 and *P. phragmitetus* was probably introduced during the previous surgery. In 1997, a male patient appeared fever after urinary catheterization, and then *P. phragmitetus* was identified in blood cultures (Jenks and Shaw, 1997). The patient had clinically important improvement after antibiotic therapy and removal of the catheter, but the patient developed fever again after recatheterization. The infection of *P. phragmitetus* was thought to be caused by insertion of the urinary catheter. In 2017, a male patient with liver abscess was diagnosed with *P. phragmitetus*. However, *P. phragmitetus* was only identified from blood cultures, but not from abscess fluid (Wang et al., 2017).

The first case of bacteriemia caused by *P. phragmitetus* in a hemodialysis patient was reported in 2020 (Gallardo et al., 2020). A male patient started hemodialysis due to impaired kidney function in Spain. After one month and a half, the patient made a trip to Cuba and did his regular hemodialysis at a local hospital. When he returned to Spain and continued hemodialysis, he developed fever during the session. The infection of *P. phragmitetus* was probably introduced during his stay in Cuba.

Highly like the case described in 2020, we reported the first hemodialysis related infection caused by *P. phragmitetus* in China. Fever occurred 4 times during or after hemodialysis in our case. However, the patient is also not engaged in environmental pollution treatment, and there is no lake near her house. Since 2022, the patient has been undergoing hemodialysis at our hospital, and there is currently no sign of fever or infection. Therefore, we speculate that *P. phragmitetus* was probably induced *via* the catheter during her regular hemodialysis at her local hospital.

Among total 5 cases of *P. phragmitetus* infection, fever appeared in 4 cases, and catheter‐related infection (CRI) was suspected to be involved in 3 cases (**Table 3**). CRI is a common but terrified complication in hemodialysis patients (Labriola, 2019). Sepsis accounts for 6.5% of deaths for patients receiving hemodialysis in USA (System USRD., 2021). Genomic sequencing showed *P. phragmitetus* is multidrug-resistant (Zhou et al., 2017), but there is no standard treatment against *P. phragmitetus*. Combining AST results from literatures and the present case, imipenem, amikacin, ciprofloxacin, and gentamicin would be ideal options for anti-infective monotherapy or combination therapy.

**Table 3:** Case and literature review.

Year	Country	Sex	Age	PMH	Clinical symptoms	Catheterizationsite	WBC(×10^9^/L)	Hb(g/L)	Creatinine(μmol/L)	Antibiotics	Ref.
1988	Britain	Male	28	*Streptococcus sanguis* endocarditis, pulmonary autograft replacement	A low-grade fever, an enlarged cardiac silhouette	UNK	UNK	UNK	UNK	6 weeks of cefuroxime and gentamicin	(Borsodi et al., 2003)
1997	Britain	Male	76	Angina pectoris, myocardial infarctions, a balloon angioplasty for coronary artery disease	Recurrent fever and hypotension after urinary catherization.	Bladder	UNK	UNK	237	The first round: 48h of cefuroxime and gentamicin, then ciprofloxacin the second round: 10 days of ciprofloxacin and gentamicin	
2017	China	Male	44	Pain in the right upper abdomen for 14 days	A large abscess within the right liver lobe	Liver	17	112	UNK	Metronidazole and cefodizime	
2020	Spain	Male	61	Hypertension, impaired kidney function, anemia, travel to Cuba	Fever after hemodialysis	Right internal jugular vein	7.56	90	589.6	Prior to AST: vancomycin and gentamicin, post AST: ciprofloxacin	
Present case	China	Female	68	End-stage renal disease, COPD, hypertension, hypothyroidism, rectal resection	Recurrent fever after hemodialysis	Right internal jugular vein	4.25	102	958.3	Prior to AST: demethylvancomycin, post AST: cefoperazone/sulbactam, gentamicin and cefdinir	

PMH, past medical history; WBC, white blood cell; UNK, unknown; AST, antimicrobial susceptibility test; COPD, chronic obstructive pulmonary disease.

## Data Availability Statement

The original contributions presented in the study are included in the article/**Supplementary Material**, further inquiries can be directed to the corresponding author/s.

## Ethics Statement

The studies involving human participants were reviewed and approved by the Medical Ethics Committee of the Central Hospital of Wuhan. The patients/participants provided their written informed consent to participate in this study.

## Author Contributions

RT, JW, and YZ collected the samples, performed the laboratory analyses, and wrote the manuscript, KW performed CT image analysis, HW revised the manuscript, ZL designed and revised the manuscript. All authors read and approved the final manuscript.

## Funding

This work was supported by the discipline construction program (2021XK071) of the Central Hospital of Wuhan.

## Conflict of Interest

The authors declare that the research was conducted in the absence of any commercial or financial relationships that could be construed as a potential conflict of interest.

## Publisher’s Note

All claims expressed in this article are solely those of the authors and do not necessarily represent those of their affiliated organizations, or those of the publisher, the editors and the reviewers. Any product that may be evaluated in this article, or claim that may be made by its manufacturer, is not guaranteed or endorsed by the publisher.
