# Supplementary material for: Hemodialysis catheter-related infection caused by Pannonibacter phragmitetus: a rare case report in China
Source: Front Cell Infect Microbiol. 2022 Jul 22;12:926154. doi: 10.3389/fcimb.2022.926154 (PMC9362148; doi:10.3389/fcimb.2022.926154)
Supplement: Supplementary file 2 [file Image_1.pdf]

Suppl. Figure 1

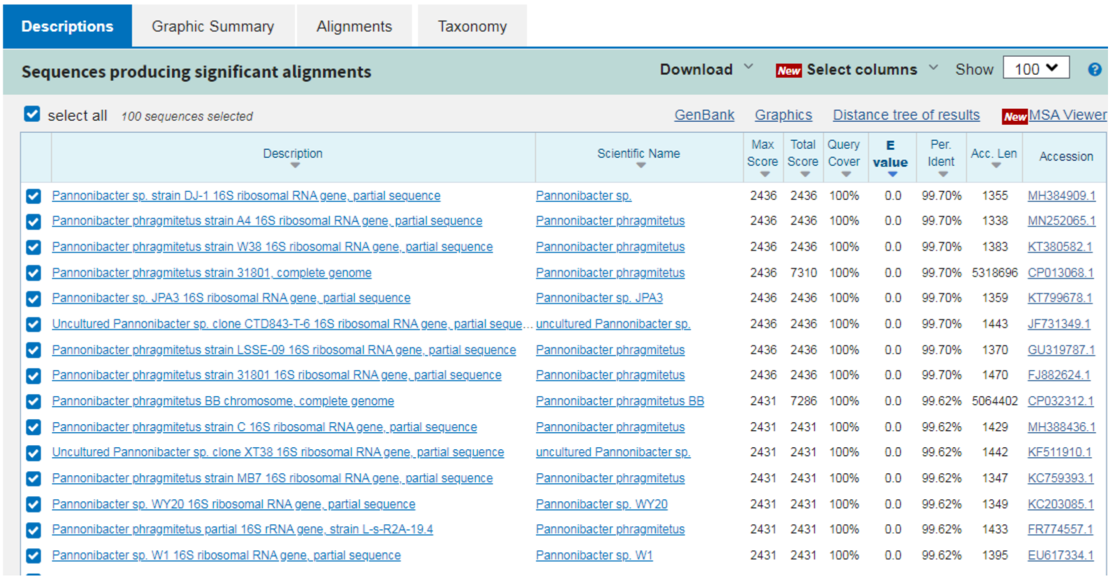

Description	Scientific Name	Max Score	Total Score	Query Cover	E value	Per. Ident	Acc. Len	Accession
Pannonibacter sp. strain DJ-1 16S ribosomal RNA gene, partial sequence	Pannonibacter sp.	2436	2436	100%	0.0	99.70%	1355	MH384909.1
Pannonibacter phragmitetus strain A4 16S ribosomal RNA gene, partial sequence	Pannonibacter phragmitetus	2436	2436	100%	0.0	99.70%	1338	MN252065.1
Pannonibacter phragmitetus strain W38 16S ribosomal RNA gene, partial sequence	Pannonibacter phragmitetus	2436	2436	100%	0.0	99.70%	1383	KT380582.1
Pannonibacter phragmitetus strain 31801, complete genome	Pannonibacter phragmitetus	2436	7310	100%	0.0	99.70%	5318696	CP013068.1
Pannonibacter sp. JPA3 16S ribosomal RNA gene, partial sequence	Pannonibacter sp. JPA3	2436	2436	100%	0.0	99.70%	1359	KT799678.1
Uncultured Pannonibacter sp. clone CTD843-T-6 16S ribosomal RNA gene, partial seque...	uncultured Pannonibacter sp.	2436	2436	100%	0.0	99.70%	1443	JF731349.1
Pannonibacter phragmitetus strain LSSE-09 16S ribosomal RNA gene, partial sequence	Pannonibacter phragmitetus	2436	2436	100%	0.0	99.70%	1370	GU319787.1
Pannonibacter phragmitetus strain 31801 16S ribosomal RNA gene, partial sequence	Pannonibacter phragmitetus	2436	2436	100%	0.0	99.70%	1470	FJ882624.1
Pannonibacter phragmitetus BB chromosome, complete genome	Pannonibacter phragmitetus BB	2431	7286	100%	0.0	99.62%	5064402	CP032312.1
Pannonibacter phragmitetus strain C 16S ribosomal RNA gene, partial sequence	Pannonibacter phragmitetus	2431	2431	100%	0.0	99.62%	1429	MH388436.1
Uncultured Pannonibacter sp. clone XT38 16S ribosomal RNA gene, partial sequence	uncultured Pannonibacter sp.	2431	2431	100%	0.0	99.62%	1442	KF511910.1
Pannonibacter phragmitetus strain MB7 16S ribosomal RNA gene, partial sequence	Pannonibacter phragmitetus	2431	2431	100%	0.0	99.62%	1347	KC759393.1
Pannonibacter sp. WY20 16S ribosomal RNA gene, partial sequence	Pannonibacter sp. WY20	2431	2431	100%	0.0	99.62%	1349	KC203085.1
Pannonibacter phragmitetus partial 16S rRNA gene, strain L-s-R2A-19.4	Pannonibacter phragmitetus	2431	2431	100%	0.0	99.62%	1433	FR774557.1
Pannonibacter sp. W1 16S ribosomal RNA gene, partial sequence	Pannonibacter sp. W1	2431	2431	100%	0.0	99.62%	1395	EU617334.1

Blast of 16S rDNA sequencing results
